# Influences of *Oldenlandia diffusa* on the CYP450 Activities in Rats Using a Cocktail Method by UHPLC-MS/MS

**DOI:** 10.1155/2018/1467143

**Published:** 2018-04-03

**Authors:** Yiping Lin, Yanli Wei, Xiaoxia Hu, Meilling Wu, Xiaoqian Ying, Mingxing Ding

**Affiliations:** ^1^Department of Pharmacology, Jinhua Polytechnic, Zhejiang, China; ^2^Central Hospital of Jinhua, Zhejiang, China

## Abstract

*Oldenlandia diffusa* has been used to treat various cancers. Cytochrome P450, a drug metabolic enzyme, might be influenced by herbal medicine. Currently, the problem that remains is the effective treatment in drug-drug interaction situation. Potential influences of *Oldenlandia diffusa* were elucidated on the CYP450 activities in rats using a cocktail method. Blood samples were precipitated by acetonitrile. Quantitative determination of target test object was done by ultra-performance liquid chromatography tandem mass spectrometry detection. Influences of *oldenlandia diffusa* on the activities of five CYP450 subtypes in rats were evaluated by five specific probe drugs (phenacetin for CYP1A2, omeprazole for CYP2C19, tolbutamide for CYP2C9, metoprolol for CYP2D6, and midazolam for CYP3A4) according to the pharmacokinetic parameters changes. No statistically significant difference (*P* > 0.05) in pharmacokinetic behaviors can be observed in the five probe drugs. There is a potential guidance on clinical drug combination with *Oldenlandia diffusa*. *Oldenlandia diffusa* in compound preparation showed well security.

## 1. Introduction

*Oldenlandia diffusa*, an herbal medicine, has a very important medicinal role used as a traditional oriental medicine for inflammatory and infectious diseases, such as pneumonia, appendicitis, and urinary tract infections [1, 2]. Moreover, previous studies had been reported that *Oldenlandia diffusa* and its major compound, ursolic acid have anticancer effects and immunomodulating activities [3, 4]. Ting et al. found that *Oldenlandia diffusa* had a promising treatment for hepatocellular carcinoma, colorectal cancer, and breast cancer [5–7].

Cytochrome P450 (CYP450), the most important drug metabolic enzyme superfamily, plays major roles in the metabolism of a variety of drugs, other xenobiotics and endogenous molecules [8–10]. Among many CYPs isoforms, human CYP1A2, CYP2C9, CYP2D6, CYP2C19, and CYP3A4 are the major CYP450 isoforms that metabolize over 90% of the clinical drugs [11, 12]. Clinically, induction or inhibition of the CYP activities could influence the pharmacokinetics of drugs, resulting in unexpected and even serious clinical drug-drug interactions (DDIs) [13, 14]. Especially, enzyme inhibition by inhibitor drugs could lead to an increase in plasma concentrations of another drug, thus increasing the risk of adverse drug reactions (ADRs) [15]. It is essential to research the inhibition or induction of CYPs in order to predict the potential DDIs and thereby avoiding the occurrence of adverse events. The cocktail approach has become one of the basic analytical tools to evaluate DDIs in vivo. Kinds of compound specially catalyzed by each CYP isoform, known as probe drugs, have been widely used to assess various individual CYP450 activities in this approach [16, 17].

For determining the safety of clinical combination use, the influences of *oldenlandia diffusa* on the activities of five CYP450 subtypes in rats is evaluated according to the pharmacokinetic parameters changes using five specific probe drugs (phenacetin for CYP1A2, omeprazole for CYP2C19, tolbutamide for CYP2C9, metoprolol for CYP2D6, and midazolam for CYP3A4); compare *oldenlandia diffusa*-treated groups with control group. A sensitive and specific ultra-performance liquid chromatography tandem mass spectrometry (UHPLC-MS/MS) method was used to simultaneously quantify the five probe drugs concentration by a single-run process.

## 2. Materials and Methods

### 2.1. Chemicals and Reagents

Phenacetin (purity > 99%), omeprazole (purity > 98%), tolbutamide (purity > 98%), and metoprolol (purity > 99%) were purchased from Toronto Research Chemicals Inc. (Toronto, Ontario, Canada). Midazolam (purity > 99%) was obtained from Tokyo Chemical Industry Co., Ltd. (Tokyo, Japan). Carbamazepine (IS) was purchased from J&K Chemical. (Shanghai, China); *Oldenlandia diffusa* (purity > 99%) was purchased from Shanghai Canspec Scientific Instruments Co., Ltd. (Shanghai, China). Formic acid (analytical reagent grade) was purchased from Sigma-Aldrich (St. Louis, MO, USA). HPLC-grade methanol and acetonitrile (ACN) were purchased from Merck Company (Darmstadt, Germany). Ultrapure water was produced in a Milli-Q system (Millipore, Bedford, MA, USA). All other chemicals and solvents were at analytical grade level.

### 2.2. Instrument and Conditions

Chromatographic separation was performed on an Agilent UHPLC unit (Agilent Corporation, MA, USA) with a ZORBAX Eclipse Plus C18 column (1.8 *μ*m, 2.1 × 50 mm, I.D. Agilent Corporation, MA, USA) maintained at 30°C. The initial mobile phase consisted of solvent A (0.1% formic acid in water) and solvent B (acetonitrile) with gradient elution as follows: 30% B (0–0.3 min), 30–50% B (0.3–1.3 min), 50–95% B (1.3–1.8 min), and 95–95% B (1.8–2.8 min). The flow rate was 0.40 mL/min. The injection volume was 5 *μ*L. The subsequent reequilibration time was 1.5 min.

The mass spectrometric detection was performed on Agilent 6420 triple-quadrupole mass spectrometer equipped with an electrospray ionization source (Agilent Corporation, MA, USA) in a positive mode. Quantitative analysis was performed in the multiple reaction monitoring (MRM) mode. The Agilent 6420 Quantitative Analysis version B.07.00 analyst data processing software (Agilent Corporation, MA, USA) was used for instrument operation and data acquisition. The precursor-product ion pairs used for the MRM detection and MS parameters are listed in Table 1.

### 2.3. Method Validation

To quantify concentration of five substances, calibration curves were established. Standard of phenacetin, tolbutamide, omeprazole, midazolam, and IS (1 mg/ml) were prepared by methanol. Concentration of each probe drugs was 0.05, 0.1, 0.25, 0.5, 1, 2.5, 5, 10 *µ*g/ml.

### 2.4. Pharmacokinetic Study

Male Sprague–Dawley (SD) rats (200–220 g) were purchased from Laboratory Animal Center of Wenzhou Medical University (Wenzhou, China). All rats were fasted for 12 h while water was not limited and used to collect 0.5 mL blank blood samples before administration. *Oldenlandia diffusa* was dissolved by physiological saline. Sixteen rats were randomly divided into two groups: the *oldenlandia diffusa* group (*n*=8) and the control group (*n*=8). All experimental procedures and protocols were reviewed and approved by the Animal Care and Use Committee of Wenzhou Medical University and were in accordance with the Guide for the Care and Use of Laboratory Animals. The control group was given physiological saline by oral administration, while the *oldenlandia diffusa* group was given *oldenlandia diffusa* (200 mg/kg) by oral administration every day for 7 days. In 7 days, rats were normally fed, except the last day which needed fasting. After 7 days, all rats were given a cocktail solution by oral administration containing five probe drugs: phenacetin (20 mg/kg), tolbutamide (1 mg/kg), omeprazole (20 mg/kg), metoprolol (20 mg/kg), and midazolam (20 mg/kg). Blood samples (0.3 ml) were collected from the tail vein at 0.167, 0.5, 1, 1.5, 2, 3, 4, 6, 9, 12, and 24 h following the experiment. The samples were immediately centrifuged at 13,000 rpm for 10 min, and the plasma was stored at −20°C.

### 2.5. Plasma Sample Preparation

Each of 100 *μ*l prepared plasma was added into 1.5 ml tubes that filled with 300 *µ*l ACN, 30 *μ*l IS (1 *μ*g/ml). After shocking through vortex (QL-901, Kylin–Bell Lab instrument) for 2 min, mixture was centrifuged using 13,000 rpm for 10 min. 100 *μ*l supernatant was diluted by 100 *μ*l ultra-water. And 5 *μ*l was injected into UHPLC-MS/MS for analysis. Other working solutions were also progressed like plasma sample.

### 2.6. Statistics and Analysis

Plasma probe drug concentration versus time data for each rat was analyzed by DAS software (version 3.0). The main pharmacokinetic parameters of the *oldenlandia diffusa* group and control group were analyzed using SPSS l8.0 statistical software. *P* < 0.05 was considered statistically significant.

## 3. Results and Discussion

### 3.1. UHPLC-MS/MS

**Figure 1:**
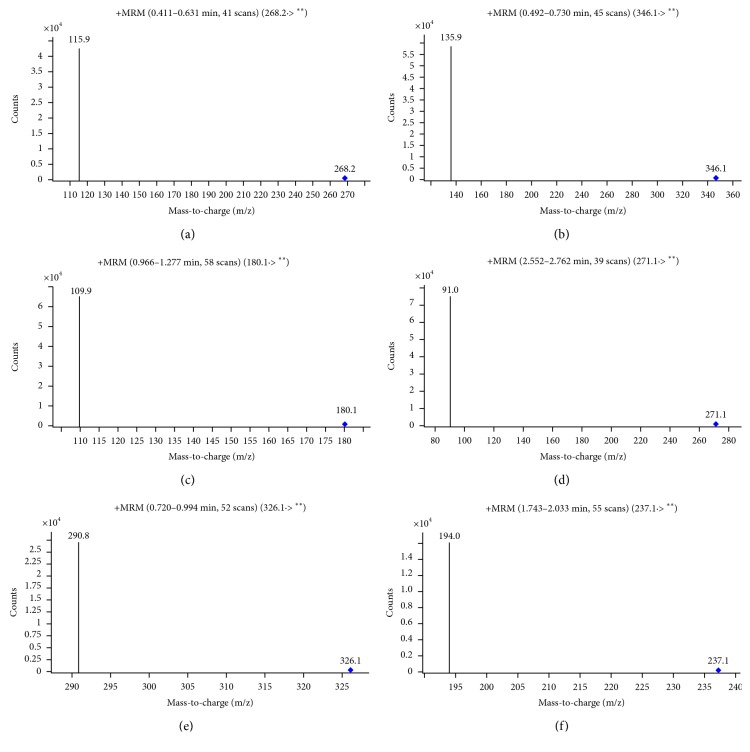
Parent and daughter ions for six substances: (a) metoprolol; (b) omeprazole; (c) phenacetin; (d) tolbutamide; (e) midazolam; (f) IS.

Figure 1 showed parent and daughter ions for each probe drugs. The post time was 0.891 min (phenacetin), 2.566 min (tolbutamide), 0.478 min (omeprazole), 0.504 min (midazolam), 0.400 min (metoprolol), and 1.588 min (IS). They had a better separation effect.

### 3.2. Pharmacokinetics

**Figure 2:**
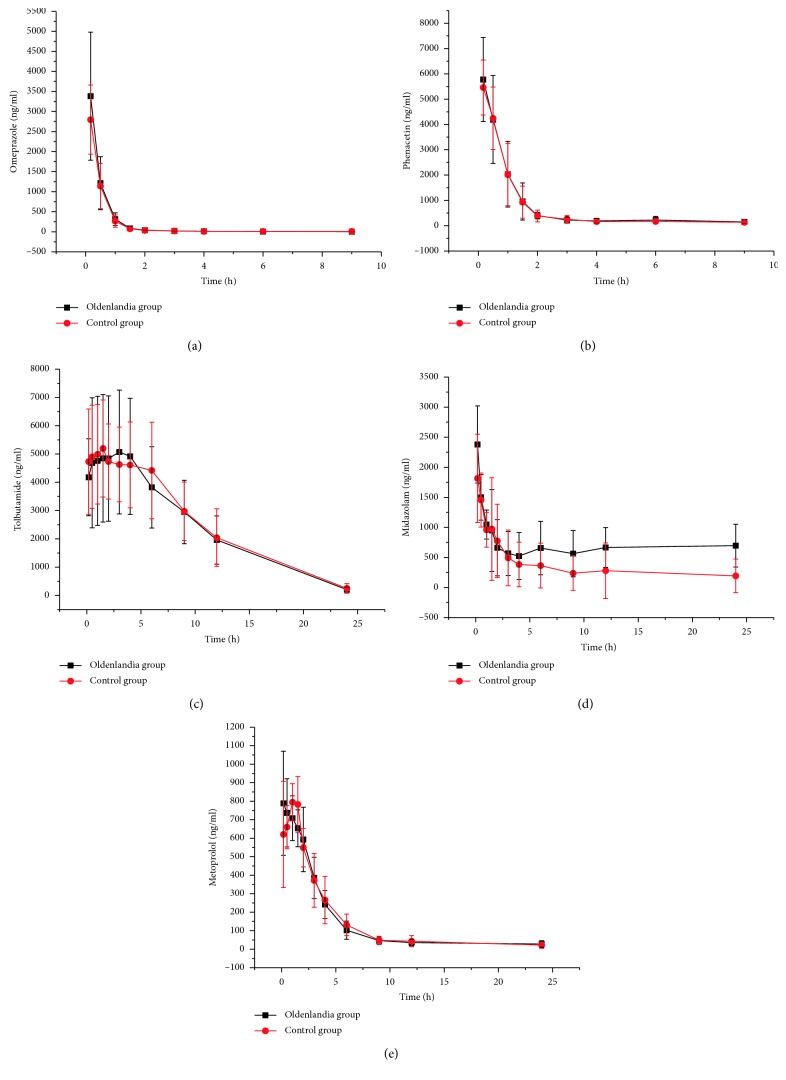
The mean plasma concentration-time curves of (a) omeprazole, (b) phenacetin, (c) tolbutamide, (d) midazolam, and (e) metoprolol.

The method was applied to the pharmacokinetic study of five probe drugs in rats. The mean plasma concentration-time curves are shown in Figure 2. The main pharmacokinetic parameters after administration of phenacetin, tolbutamide, omeprazole, metoprolol, and midazolam from noncompartment model analysis are summarized in Table 2.

From Table 2, in the experiment for the *oldenlandia diffusa* and control groups, there was insignificant difference (*P* > 0.05) in pharmacokinetic behaviors could be observed for phenacetin, tolbutamide, omeprazole, metoprolol, and midazolam. Results of the present study showed that *oldenlandia diffusa* might not affect the activity of CYP1A2, CYP2C9, CYP2C19, CYP2D6, and CYP3A4 in rats. CYP450 was responsible for ∼90% clinical drugs, including anti-inflammatory, cardiovascular, and cancer drugs [18]. Oldenlandia diffuse had a possibility to participate in drug combination. And according to Wu et al.'s research, classical five probe drugs were selected [19]. These *Oldenlandia diffusa* was applied as a traditional Chinese medicine in Asia which was mainly distributed in south of the Yangtze River [20]. There were many chemical compositions in *Oldenlandia diffusa*, but flavonoids, anthraquinone, and iridoid glucosides were mainly active pharmacological effect [21, 22]. The further research needs to know if these monomers have interaction on CYP that pharmacokinetics of five probe drugs had negligible change. And whether *oldenlandia diffusa* affects the drugs through other metabolic pathways or not is still an issue.

## 4. Conclusion

This research showed that *oldenlandia diffusa* had no effect on CYP1A2, CYP2C9, CYP2C19, CYP2D6, and CYP3A4. And there was a potential guidance on clinical drug combination that *oldenlandia diffusa* might be considered as a safety combination drug with five CYPs metabolism drugs in clinical. But the interaction between *oldenlandia diffusa* and drugs which not involved in CYP450 metabolism still need attention.

## Figures and Tables

**Table 1:** Mass spectrometry information for each probe drugs.

Compound	Precursor ion (m/z)	Product ion (m/z)	Collision energy (V)	Fragmentor
Phenacetin	180.1	109.9	24	122
Omeprazole	346.12	135.9	44	108
Tolbutamide	271.11	91	36	120
Metoprolol	268.19	115.9	17	130
Midazolam	326.09	290.8	44	170
Carbamazepine	237.1	194	18	140

**Table 2:** The pharmacokinetics parameters of phenacetin, omeprazole, tolbutamide, midazolam, and metoprolol in rat plasma after administration of *oldenlandia diffusa*.

Probe drug	Pharmacokinetics parameters	Control group	*Oldenlandia diffusa*-treated group
Phenaceton	AUC (0–t) (mg/L∗h)	6.00 ± 1.60	6.28 ± 1.81
	AUC (0–∞) (mg/L∗h)	7.37 ± 2.12	13.1 ± 9.31
	t1/2 (h)	5.98 ± 3.58	24.67 ± 39.93
	Tmax (h)	0.22 ± 0.14	0.17 ± 0.00
	Vz/F (L/kg)	23.3 ± 10.5	34.6 ± 34.8
	CLz/F (L/h/kg)	2.90 ± 0.80	1.98 ± 0.82
	Cmax (mg/L)	5.78 ± 1.66	5.72 ± 0.76
Omeprazole	AUC (0–t) (mg/L∗h)	1.45 ± 0.522	1.63 ± 0.754
	AUC (0–∞) (mg/L∗h)	1.48 ± 0.528	1.64 ± 0.759
	t1/2 (h)	3.28 ± 1.47	1.86 ± 1.08
	Tmax (h)	0.17 ± 0.00	0.17 ± 0.00
	Vz/F (L/kg)	77.8 ± 56.0	33.2 ± 12.0
	CLz/F (L/h/kg)	16.1 ± 9.20	15.5 ± 9.03
	Cmax (mg/L)	2.80 ± 0.862	3.38 ± 1.60
Tolbutamide	AUC (0–t) (mg/L∗h)	60.2 ± 22.7	58.2 ± 22.3
	AUC (0–∞) (mg/L∗h)	61.9 ± 23.7	62.5 ± 26.3
	t1/2 (h)	4.21 ± 0.69	5.32 ± 1.84
	Tmax (h)	2.00 ± 1.67	2.27 ± 1.21
	Vz/F (L/kg)	0.11 ± 0.03	0.13 ± 0.05
	CLz/F (L/h/kg)	0.02 ± 0.01	0.02 ± 0.01
	Cmax (mg/L)	5.29 ± 1.78	5.45 ± 2.29
Midazolam	AUC (0–t) (mg/L∗h)	8.24 ± 8.25	13.8 ± 9.57
	AUC (0–∞) (mg/L∗h)	36.1 ± 83.0	77.9 ± 81.7
	t1/2 (h)	29.3 ± 63.4	60.0 ± 68.3
	Tmax (h)	0.88 ± 0.82	0.17 ± 0.00
	Vz/F (L/kg)	28.0 ± 17.2	44.1 ± 80.2
	CLz/F (L/h/kg)	4.93 ± 4.69	3.42 ± 4.44
	Cmax (mg/L)	2.00 ± 0.608	2.38 ± 0.641
Metoprolol	AUC (0–t) (mg/L∗h)	3.33 ± 0.798	3.16 ± 0.576
	AUC (0–∞) (mg/L∗h)	3.54 ± 1.05	3.94 ± 1.53
	t1/2 (h)	6.80 ± 5.09	15.7 ± 18.5
	Tmax (h)	0.920 ± 0.510	0.690 ± 0.630
	Vz/F (L/kg)	53.9 ± 30.8	89.7 ± 79.3
	CLz/F (L/h/kg)	6.06 ± 1.58	5.72 ± 1.95
	Cmax (mg/L)	0.865 ± 0.142	0.933 ± 0.172
